# The relationship between breast density, age, and mammographic lesion type among Chinese breast cancer patients from a large clinical dataset

**DOI:** 10.1186/s12880-021-00565-9

**Published:** 2021-03-08

**Authors:** Yu Ji, Boxin Li, Rui Zhao, Ying Zhang, Junjun Liu, Hong Lu

**Affiliations:** 1 Department of Breast Imaging, Tianjin Medical University Cancer Institute and Hospital, Tianjin, China; 2 National Clinical Research Center for Cancer, Key Laboratory of Cancer Prevention and Therapy, Tianjin’s Clinical Research Center for Cancer, Key Laboratory of Breast Cancer Prevention and Therapy, Tianjin Medical University, Ministry of Education, Tianjin, China

**Keywords:** Breast cancer, Breast density, Age, Mammographic lesion type, Chinese women

## Abstract

**Background:**

The purpose of this study was to investigate the relationship between breast density, age, and mammographic lesion type among Chinese breast cancer patients included in a large clinical dataset.

**Methods:**

A review of mammographic images acquired between July 2014 and June 2017 from a total of 9716 retrospectively registered breast cancer patients was conducted. Mammographic breast density was defined according to the American College of Radiology Breast Imaging Reporting and Data System (ACR BI-RADS) 4-class density rating. Mammographic lesion types were defined according to the ACR BI-RADS, including mass, mass with calcifications, calcifications, architectural distortion/asymmetries, and architectural distortion/asymmetries with calcifications. Three experienced breast radiologists interpreted all mammograms. The chi-square (χ^2^) test and Pearson correlation analyses were performed to assess the relationship between breast density, age, and mammographic lesion type.

**Results:**

A significant inverse relationship was observed between the BI-RADS breast density rating given by radiologists and patient age (r = − 0.521, *p* < 0.01). The breast density distribution in breast cancer patients from China reversed at the age of 55 years, and exhibited one age peak in the age 55–59 year group. The percentage of lesions with calcifications decreased with increasing age (*p* < 0.01), and increased with increasing breast density (*p* < 0.01).

**Conclusions:**

In general, we identified a relationship between patient breast density, age, and mammographic lesion type. This finding may provide a basis for clinical diagnoses and support development of breast cancer screening programs in China.

## Background

Breast cancer is the most common cancer in women worldwide [1]. Advances in mammography have facilitated effective clinical diagnoses and screening programs. In 1976, Wolfe initially proposed a relationship between mammographic parenchymal patterns and the risk of breast cancer [2, 3]. The association between increased breast density and an increased risk for breast cancer is not well understood. However, it has previously been shown that increased breast density may limit the sensitivity of mammography for breast cancer [4].

Many investigators have attempted to identify why some cancers are missed by mammography. Lehman et al. [5] reported that breast density might be a predictor of mammographic performance. Bird et al. [6] found a greater percentage of missed cancers in dense breasts. However, dense breasts were cited as a factor for missed lesions in cases of masses more often than cases of calcifications [4]. These results suggested that factors associated with missed detection of cancers differed with respect to the types of lesions. Moreover, the compromised accuracy of mammographic screening generally appeared to be more relevant to younger women. Several studies reported that age was an important independent predictor for sensitivity and specificity in mammographic screening. Overall, a higher proportion of young women tended to have dense breast tissue compared to older women, limiting the diagnostic sensitivity of the examination [7, 8].

A better understanding of the relationship between breast density, age, and mammographic lesion type would help identify the reasons why some cancers are missed by mammography. Studies investigating this relationship have been performed in western countries, but similar research performed in a large Chinese breast cancer patient population is lacking. Therefore, the purpose of this study was to investigate the relationship between breast density, age, and mammographic lesion type among Chinese breast cancer patients.

## Methods

### Database

The mammography dataset used in this study was retrospectively collected and de-identified, and thus the study was deemed exempt by the Ethics Committee at Tianjin Medical University Cancer Institute and Hospital (EK2020105). The mammographic images from 9716 patients with breast cancer confirmed by histopathology that underwent diagnostic mammography examinations between July 2014 and June 2017, in the department of breast imaging diagnosis of Tianjin Medical University Cancer Institute and Hospital. The ages of the 9716 patients included in the study ranged from 19 to 93 years, with an average of 54.3 ± 11.2 years, and a median of 54 years.

### Classification of mammograms

Craniocaudal and mediolateral oblique views of bilateral mammograms were obtained using full-field digital mammography systems (Senographe 2000D, General Electric Company, USA; and Selenia Digital Mammography, Hologic Inc., USA).

**Fig. 1:**
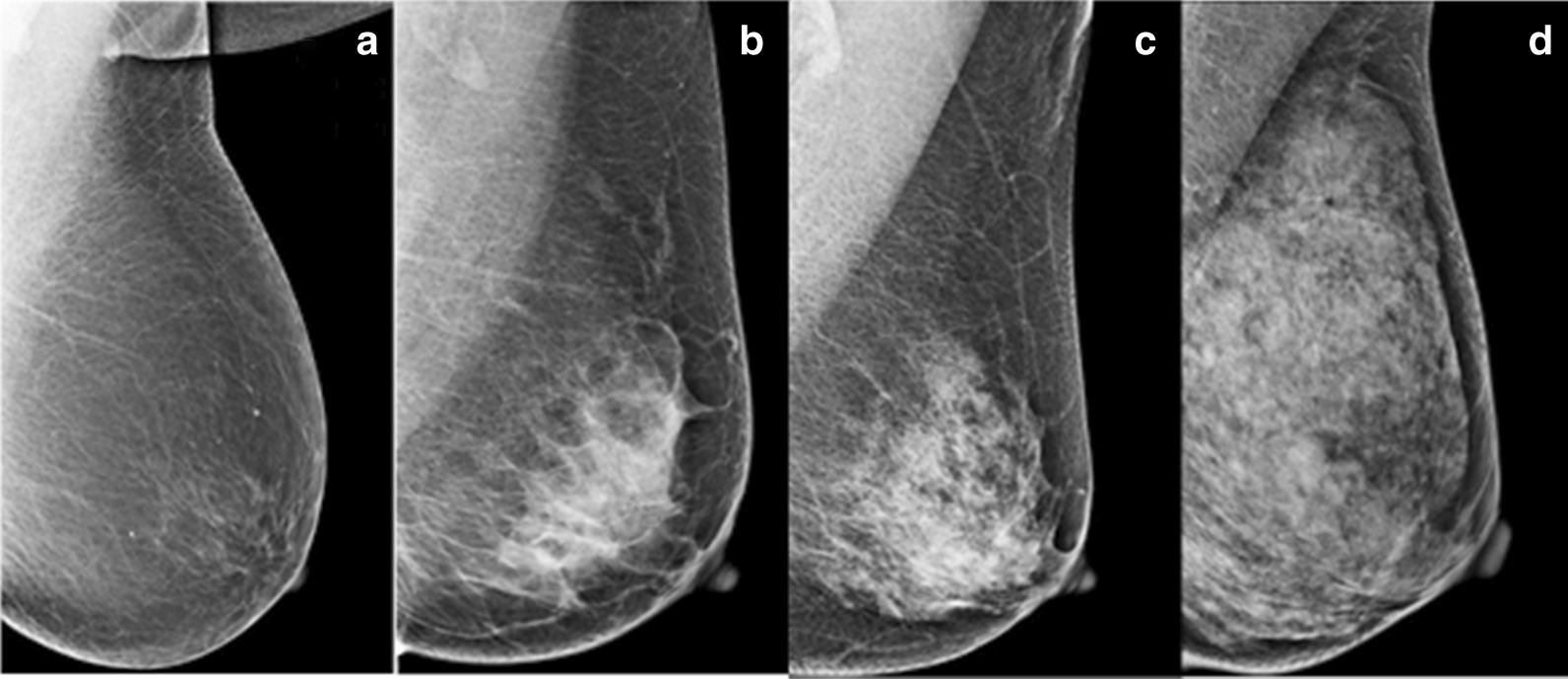
BI-RADS Density Types in Four Types. **a** almost entirely fat (Class A), **b** scattered fibroglandular densities (Class B), **c** heterogeneously dense (Class C), **d** extremely densities (Class D)

Mammographic breast density was defined according to the American College of Radiology Breast Imaging Reporting and Data System: Class A, almost entirely fat; Class B, scattered fibroglandular densities; Class C, heterogeneously dense; and Class D, extremely dense (Fig. 1). Classes A and B were defined as low density, and classes C and D were defined as high density. In addition, mammographic lesion types were defined according to the ACR BI-RADS, including mass, mass with calcifications, calcifications, architectural distortion/asymmetries, and architectural distortion/asymmetries with calcifications. All mammograms were interpreted by three experienced breast radiologists.

### Statistical analysis

The chi-square (χ^2^) test and Pearson correlation analyses were used to assess the relationship between age, density, and mammographic lesion type. To evaluate the significance of a linear association, we conducted the analysis of variance with age as a continuous variable. All analyses were performed with SPSS software (version 19.0, SPSS, IBM Corp., Armonk, NY, USA). The reported *p* values were two-sided. A *p* value < 0.05 was set as the threshold for statistical significance.

## Results

**Fig. 2:**
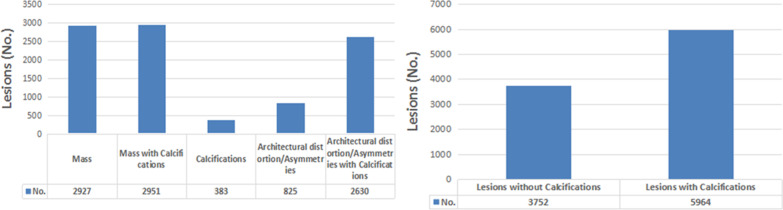
Bar graph showing patient age and number (NO) of breast cancer patients. Age distribution of breast cancer cases. The age peak group was the 55–59 year group

The distribution of the study population according to breast density and age is shown in Table 1. A significant association between breast density and age is evident. Increasing age was associated with decreasing breast density (r = − 0.521, *p* < 0.01), with a frequency of heterogeneously or extremely dense (class C or D) breast tissue of 84.62%, 85.32%, 81.68%, 82.21%, 81.14%, 77.08%, 62.29%, 42.97%, 30.17%, 22.12%, 17.09%, 11.18%, 6.59%, 14.29%, and 0.00% in the age groups of 19–24, 25–29, 30–34, 35–39, 40–44, 45–49, 50–54, 55–59, 60–64, 65–69, 70–74, 75–79, 80–84, 85–89, and 90–94 years, respectively. In addition, we observed that more women were diagnosed with breast cancer in the 55–59 year age group (Fig. 2).

**Table 1:** Breast density of 9716 breast cancer patients categorized by age

Age (years)	Breast density of 9716 breast cancer patients categorized by age^a^				
	No. of patients	Breast tissue density			
		Predominantly fat	Scattered fibroglandular	Heterogeneously dense	Extremely dense
19–24	13	0 (0.00%)	2 (15.38%)	5 (38.46%)	6 (46.15%)
25–29	109	0 (0.00%)	16 (14.68%)	64 (58.72%)	29 (26.61%)
30–34	262	5 (1.91%)	43 (16.41%)	161 (61.45%)	53 (20.23%)
35–39	511	8 (1.57%)	88 (17.22%)	355 (69.47%)	60 (11.74%)
40–44	1039	13 (1.25%)	183 (17.61%)	741 (71.32%)	102 (9.82%)
45–49	1383	32 (2.31%)	285 (20.61%)	988 (71.44%)	78 (5.64%)
50–54	1575	100 (6.35%)	494 (31.37%)	935 (59.37%)	46 (2.92%)
55–59	1720	232 (13.49%)	749 (43.55%)	725 (42.15%)	14 (0.81%)
60–64	1422	327 (23.00%)	666 (46.84%)	424 (29.82%)	5 (0.35%)
65–69	782	281 (35.93%)	328 (41.94%)	171 (21.87%)	2 (0.26%)
70–74	474	201 (42.41%)	192 (40.51%)	80 (16.88%)	1 (0.21%)
75–79	313	159 (50.80%)	119 (38.02%)	35 (11.18%)	0 (0.00%)
80–84	91	43 (47.25%)	42 (46.15%)	5 (5.49%)	1 (1.10%)
85–89	21	12 (57.14%)	6 (28.57%)	3 (14.29%)	0 (0.00%)
90–94	1	1 (100.00%)	0 (0.00%)	0 (0.00%)	0 (0.00%)
All ages	9716	1,414 (14.55%)	3213 (33.07%)	4692 (48.29)	397 (4.09)

Numbers within parentheses are percentages

^a^Chi-square test for difference in proportions: (χ^2^ = 1857.01, *p* < 0.05)

**Fig. 3:**
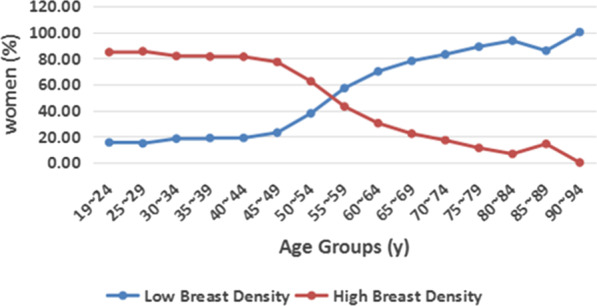
Bar graphs showing mammographic lesion types and number (NO) of breast cancer lesions

The distribution of the study population by mammographic lesion type is shown in Fig. 3. The frequencies of mass, mass with calcifications, calcifications, architectural distortion/asymmetries, and architectural distortion/asymmetries with calcifications were 2927, 2951, 383, 825, and 2630, respectively. There were 5964 mammographic lesions with calcifications, and 3,753 mammographic lesions without calcifications.

**Fig. 4:**
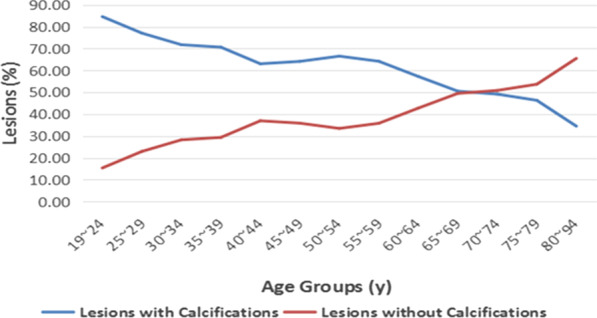
Line chart showing patient age, classifications, and breast categories. Mammographic breast density decreases with increasing age. There is a significant inverse relationship between age and breast density (r = − 0.523, *p* < 0.01)

**Fig. 5:**
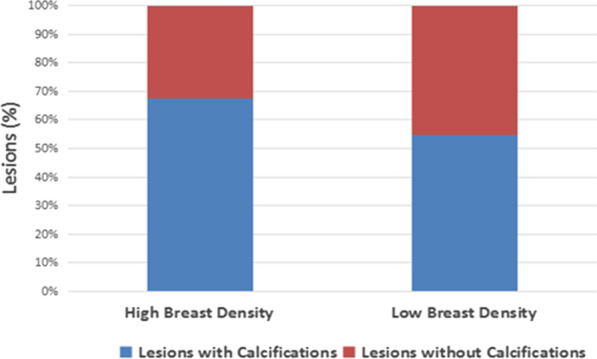
Line chart showing patient age and lesions with calcifications. The percentage of lesions with calcifications decreases with increasing age (*p* < 0.01)

**Fig. 6:**
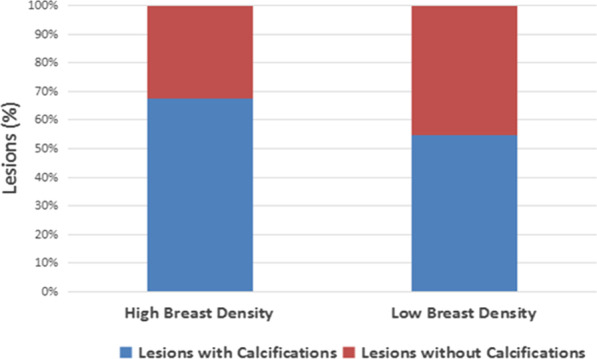
Bar graph showing breast density and lesions with calcifications. The percentage of lesions with calcifications increases with increasing breast density (χ^2^ = 171.85, *p* < 0.01)

The results illustrated in Fig. 4 demonstrate that the distribution of mammographic breast density in Chinese breast cancer patients included in this study reversed at 55 years, and the difference in breast density between the two age groups mentioned above was statistically significant (*p* < 0.01). More women with fatty breasts were diagnosed with breast cancer at an older age, and more women with dense breasts were diagnosed at younger age. The distribution of the population by age and calcifications shown in Fig. 5 demonstrates that the percentage of lesions with calcifications decreased with increasing age (*p* < 0.01). The distribution of the population by breast density and calcifications shown in Fig. 6 reveals that the percentage of lesions with calcifications increased with increasing breast density (*p* < 0.01).

## Discussion

The results of this study indicated that there was a significant inverse relationship between breast density and patient age, and the percentage of lesions with calcifications was higher in dense breasts and younger women. Notably, all the cases in this pilot study had positive mammographic findings.

We found that mammographic sensitivity was 80% in women with predominantly fatty breasts and 30% in women with mammographically dense breasts [9]. It was previously demonstrated that dense tissue could obscure subtle signs of malignancy and thereby decrease the sensitivity of mammography [7]. The language of the BI-RADS lexicon accounts for this by referring to dense tissue patterns as “the breasts are heterogeneously dense, which may obscure small masses” and “the breasts are extremely dense, which lowers the sensitivity of mammography” [10]. Breast density typically decreased with increasing age and therefore younger women had more dense breasts. Some reports showed that there was an inverse effect of density on mammographic sensitivity, with a parallel age effect [11]. Similar results were observed in our research. We found that a significant inverse relationship between age and breast density existed in Chinese breast cancer patients, and that the distribution of mammographic breast density reversed at an age of 55 years. It is worth noting that in our study, we found that 62.29% of the patients in the 50–54 age group had heterogeneously or extremely dense (class C or D) breast tissue. Consequently, this finding may make mammography-based screening less attractive in China. Digital breast tomosynthesis (DBT) and magnetic resonance imaging (MRI) have demonstrated improved cancer detection in dense breasts compared to mammography. Resulting simultaneous reduction of recalls, DBT is the preferred mammographic technique for women with dense breasts. MRI provides the greatest increase in cancer detection and decreases interval cancers and late-stage disease; abbreviated techniques will reduce cost and improve availability. Understanding performance of mammography and supplemental imaging modalities is necessary to optimize screening for women with dense breasts.

Different studies have reported conflicting results on the sensitivity of mammography [12, 13]. Beyond the effects of breast density, it is possible that the proportion of lesions with calcifications versus those without calcifications included in the studies could have impacted statistical analyses. Breast density may mask nonpalpable cancers on mammograms as masses or architectural distortions, but it is less likely to mask calcifications. The accuracy of a radiologist’s interpretation of digital mammograms depends on lesion type [14]. In this study, we investigated the potential relationship between breast density and mammographic lesion type in Chinese breast cancer patients, and found that the proportion of calcified breast lesions is relatively high in dense breasts. Our study also indicated that the percentage of lesions with calcifications decreased with increasing age. Chinese women tend to have small and dense breasts, and the mean age of diagnosis for breast cancer is considerably younger than that of western women. Our results indicated that a higher proportion of young Chinese breast cancer patients had dense breasts with calcified lesions. Consequently, this finding may highlight the limitations of mammography-based screening programs in the Chinese population.

Although there are many similarities in the breast cancer screening guidelines issued by various agencies, there are still some differences. This difference mainly focuses on the age at which screening begins. The U.S. Preventive Services Task Force and the International Agency for Research on Cancer believe that the starting age for screening should be 50 years old, while the American Cancer Society believes that regular screening should be started at 45 years old for women with normal risk of breast cancer. The National Comprehensive Cancer Network recommends regular screening for women at normal risk of breast cancer from the age of 40. The National Central Cancer Registry of China reported that the peak age for breast cancer was between 55 and 59 years, as determined by existing population-based cancer registry data [15]. Similar results were also observed in our study, whereas the peak age in other Asian countries is between 45 and 50, and between 55 and 60 in Western countries [16]. It should be noted that the age ranges in these studies indicate ages at diagnosis. Our study provides a better understanding of breast cancer status in Chinese women based on patient data, and indirectly provides a reference for development of breast cancer screening guidelines suitable for existing conditions in China. Therefore, if our results verifiably reflect the current breast cancer situation in China, it may be possible to design a preliminary Chinese population-based screening program for breast cancer in economically underdeveloped areas, initially focusing on the 55–59 year age group which exhibited the highest proportion of breast cancers in our study.

In 2008, the Chinese government initiated a breast cancer screening program in rural areas and in some major cities promoting early detection and diagnosis, as well as public awareness of disease prevention. However, there is currently no nationwide screening program for breast cancer in China. One of the most important reasons for the lack of such a program is insufficient mammography equipment [17]. Breast ultrasound (US) may be more suitable for breast cancer screening in China at present since it is not limited by breast density, is portable, is less expensive, and does not involve ionizing radiation. Reports showed that cancer detection by breast US was very similar to mammography [18], particularly in premenopausal patients [19]. Our results indicated that a high proportion of cancerous lesions had calcifications. Notably, the degree of micro-calcification detection by US has been debated. Some authors claimed high sensitivity of up to 98.1% [20, 21], whereas other authors concluded that US was unsuitable for the detection of micro-calcifications [22]. Further investigations with larger study populations are necessary before US-based breast screening programs are implemented in China.

In our study, all mammograms were interpreted by three experienced breast radiologists. We acknowledge the potential for inter-reader variability in qualitative breast-density measurements and type definitions of mammographic lesions. However, we believe that variability in our results was minimized due to our specialized training and exclusive breast-imaging practice.

It should be noted that there were some limitations to our study. Several factors previously shown to correlate with breast density such as weight, nutritional status, age at first birth, parity, and menopausal status were not considered in this study.

We concluded that a relationship between breast density, age, and the type of mammographic lesion existed among Chinese breast cancer patients. This finding may provide the basis for clinical diagnoses and screening programs for breast cancer. Mammographic sensitivity may increase with decreasing breast density and increasing patient age, and depends on the lesion type, even in cases of dense breasts. Information on individual patient breast density, age, and lesion calcifications included in examination reports could support the clinical evaluation of mammographic sensitivity, and could also provide women with an increased understanding of the detection levels that mammography can offer.

## Data Availability

The datasets used and/or analyzed during the current study are available from the corresponding author on reasonable request.

## Abbreviations

ACR BI-RADS: American College of Radiology Breast Imaging Reporting and Data System
US: Ultrasound
